# Anti-carbamylated protein antibodies and skin involvement in patients with systemic sclerosis: An intriguing association

**DOI:** 10.1371/journal.pone.0210023

**Published:** 2018-12-31

**Authors:** Elvira Favoino, Marcella Prete, Serena Vettori, Addolorata Corrado, Francesco Paolo Cantatore, Gabriele Valentini, Federico Perosa

**Affiliations:** 1 Department of Biomedical Sciences and Human Oncology (DIMO), Rheumatologic and Systemic Autoimmune Diseases Unit, University of Bari Medical School, Bari, Italy; 2 Department of Clinical and Experimental Internal Medicine “F. Magrassi-A. Lanzara”, Rheumatology Section, University of Campania, Naples, Italy; 3 Department of Medical and Surgery Sciences, Rheumatology Unit, University of Foggia, Foggia, Italy; University of Naples, ITALY

## Abstract

Carbamylation is a post-translational modification that mostly affects proteins with low turnover, such as dermal proteins. Carbamylated proteins accumulate in skin in an age-dependent manner, contributing to tissue alterations. As dermis is affected by systemic sclerosis (SSc) and anti-carbamylated protein antibodies (anti-CarP Ab) are found in SSc patients, we sought to evaluate the specificity of anti-CarP Ab and their relationship with clinical parameters reflecting skin involvement in SSc. This study investigated serum samples and clinical data from 124 patients with SSc. Anti-CarP Ab were affinity purified from pooled SSc sera, and their specificity was assessed by western blotting and ELISA with carbamylated proteins from two species (human and bovine albumin; human fibrinogen). Anti-CarP Ab were measured in SSc serum samples and in 41 healthy aged-matched individuals. Affinity-purified anti-CarP Ab recognized carbamylated epitopes irrespective of the protein type or species origin. Anti-CarP Ab levels inversely correlated with the modified Rodnan skin score (mRss) (Spearman’s R = -0.32, p<0.001), independently of patients’ age. Receiver operating characteristics (ROC) analysis identified anti-CarP Ab cut-offs that best discriminated dichotomized clinical variables related to skin involvement: the only clinical variables that were significantly different between groups were mRss (p = 0.001) and scleredema (p<0.001). Low anti-CarP Ab levels were associated with worse skin involvement. Future prospective studies are needed to assess their usefulness in the clinical setting.

## Introduction

Systemic sclerosis (SSc) is a rare, multisystem, connective tissue disease of unknown etiology and pathogenesis, characterized by three interconnected pathogenic events, namely vascular abnormalities, abnormal extracellular matrix (ECM) deposition, and autoimmunity [1,2]. Though heterogeneous in its clinical manifestations, SSc causes widespread fibrosis of the skin and internal organs, leading to disability and death.

Patients with SSc present auto-antibodies (Ab) with a wide range of specificity. Circulating antinuclear Ab directed to self-antigens such as DNA topoisomerase I, RNA polymerase III, the Th/To autoantigen, and heterologous centromeric proteins (CENPs) have been detected in over 95% of patients [3], and have diagnostic and prognostic value [1,2,4]. Anti-CENP Ab sub-specificities have been described by our group as predictive of pulmonary vascular disease [5]. Other auto-Ab described in SSc, including anti-endothelial cell Ab [6–8], anti-fibroblast Ab [9,10], anti-angiotensin receptor and anti-endothelin receptor Ab [11], also appear to have clinical significance [12–16]. Another family of Ab, which seems to have clinical significance in SSc, rheumatoid arthritis (RA) and other connective tissue diseases, is directed against post-translationally modified proteins, including citrullinated and carbamylated proteins.

Citrullination is the enzymatic conversion of peptidyl-arginine into peptidyl-citrulline[17]. Carbamylation is instead a nonenzymatic modification whereby cyanate, a dissociation product of urea, reacts with peptidyl-lysine to generate homocitrulline[18,19]. Several factors trigger protein carbamylation, including high urea concentration and inflammation [20]. The extent of carbamylation depends on the number and accessibility of lysine residues in target proteins [21] and the reaction is almost irreversible [22]. Therefore, the extent of carbamylation is more apparent in proteins with long half-lives (low turnover rates), which accumulate homocitrulline residues over time. Proteins with low turnover include dermal (or tendon) elastin [23] and collagen [24], the most abundant protein in ECM.

Previous studies identified various antigens that are targeted by Ab against carbamylated proteins (anti-CarP Ab), namely albumin [25], hemoglobin [26], LDL [27], fibrinogen [28], alpha-1-anti-trypsin [29], the 78-kDa glucose-regulated protein (GRP78)[30], enolase [31], and vimentin [32]. Cross-reactivity of anti-CarP Ab from RA patients with citrullinated fetal calf serum (FCS) has been reported [19]. Even so, the specificity of these Ab has not been thoroughly investigated. Pecani et al. reported the presence of anti-CarP Ab in SSc, and showed that there is barely a difference in the level of anti-CarP Ab between SSc patients and healthy donors (p = 0.03)[21]. The possible association between these Ab and the clinical manifestations of SSc has not yet been analyzed.

Considering that dermis is one of the main targets of SSc and that dermal proteins undergo carbamylation, here we assessed whether the neo-carbamylated epitopes recognized by anti-CarP Ab in SSc are conserved among different proteins from different species and whether the levels of these Ab are associated with clinical manifestations related to skin involvement in SSc.

## Materials and methods

### Patients and clinical data

124 SSc patients who fulfilled both the 1980 ACR [33] and the 2013 ACR/EULAR [34] criteria for the classification of SSc were recruited from the Rheumatology Units of the Universities of Naples, Bari, and Foggia from 2010 to 2016. For each SSc patient, data related to sex, age at diagnosis (measured from the onset of the first Raynaud’s phenomenon), age at observation (i.e. when the patient was last seen and blood was sampled), and laboratory test results were collected. Skin involvement was evaluated as limited or diffuse according to LeRoy et al.’s criteria [35]. Disease severity was determined on the Medsger severity scale [36]. Skin involvement was measured using mRss, whereby the degree of skin thickness is measured in 17 body areas on a scale from 0 (normal) to 3 (severe), for a total score range of 0–51 [37]. Scleredema was scored as present or absent.On the basis of the presence of anti-centromere Ab (ACA) and anti-topoisomerase-I (anti-Scl70) Ab, patients were subdivided in ACA^+^ (*n* = 56) and anti-Scl70 Ab^+^ (*n* = 55).

This study was approved by the Ethics Committees of the Universities of Naples, Bari and Foggia, and written informed consent was obtained from each participant.

### Reagents and serum samples

Bovine serum albumin (BSA) and human fibrinogen (FIB) were purchased from Sigma-Aldrich. Human albumin (HA) was purchased from Kedrion Biopharma (Barga, Italy). Electrophoresis reagents were purchased from Bio-Rad Laboratories. Horseradish-peroxidase (HRP)-conjugated xeno-Ab to human IgG (Fc portion) was purchased from Jackson Immunoresearch Laboratories (Avondale, USA).

Serum samples from the 124 SSc patients and from 41 age-matched healthy blood donors (HBD) were stored at -80°C until use.

### Carbamylation of proteins

Carbamylated BSA (CarBSA), human albumin (CarHA), and human fibrinogen (CarFIB) were generated by incubating 2 mg protein in 1 ml 8 M urea, 100 mM Tris-HCl (pH 8.5), for 15 h at 61°C. After extensive dialysis against phosphate-buffered saline (PBS), carbamylated protein concentration was assessed using the BCA assay (Thermo Fisher Scientific). Success of the carbamylation procedure was verified by documenting a reduced electrophoretic mobility [24] by SDS-PAGE on 10% polyacrylamide gels under reducing conditions.

### Serological assays

SSc serum samples were screened for anti-CarP Abusing a modification of the ELISA protocol developed by Shi et al. [19]. Briefly, 96-well polyvinylchloride microtiter plates were coated with 5 μg/ml BSA in carbamylated or native form. Wells were washed once with PBS containing 0.05% Tween-20, and any free protein-binding sites were blocked with PBS containing 0.5% BSA. Wells were incubated for 4 h with serum samples (diluted 1:100 in PBS containing 0.1% BSA). One serum sample from an RA patient was used as a positive control. Bound IgG was revealed with HRP-conjugated anti-human IgG and *o*-phenylenediamine. Specific binding was determined by subtracting the background binding in BSA-coated wells from the binding in experimental wells. The levels of anti-CarP Ab in sera were expressed as a percentage of binding relative to that of the positive control. Samples were tested in duplicate, and the experiment was performed at least 3 times. Ten serum samples with highest titer were pooled and used for affinity purification of anti-CarP Ab. The specificity of anti-CarP Ab was assessed by ELISA in binding and inhibition assays as previously described [38].

### Affinity purification of human anti-CarP Ab

BSA (10 mg/ml) and CarBSA (10 mg/ml) were conjugated separately to Affi-Gel 15 (Bio-Rad Laboratories) following the manufacturer’s instructions. Pooled serum (15 ml) from SSc patients having high binding avidity for CarBSA was diluted 5-fold in PBS, and subjected to affinity chromatography on BSA-conjugated Affi-Gel 15 column to adsorb BSA-specific Ab. Then, the nonbinding fraction was passed several times over a CarBSA-conjugated column; bound IgG were eluted with glycine-HCl (pH 2.3) and dialyzed overnight against PBS diluted 1:10. Anti-CarP Ab were concentrated 10x by lyophilization and their concentration was determined by BCA protein assay. The purity of anti-CarBSA Ab was assessed by SDS-PAGE under non-reducing conditions [38].

### Western blotting

Carbamylated and native proteins were resolved by 10% SDS-PAGE under reducing conditions and transferred onto polyvinylidene fluoride (PVDF) Immobilon P filters (Millipore), previously soaked in absolute methanol. Free protein-binding sites were blocked by a 2 h incubation in PBS containing 5% nonfat dry milk. Then, filters were incubated with 2 μg/ml affinity-purified anti-CarP Ab overnight at 4°C. After extensive washing, filters were incubated with HRP-conjugated xeno-Ab to the Fc portion of human IgG (1 h, room temperature), and developed using the Clarity Western ECL Substrate (Bio-Rad).

### Statistical analyses

The significance of differences between the medians of continuous variables was determined by the nonparametric Kruskal-Wallis H test. Spearman’s rho test was used for correlation analysis between two continuous variables. Receiver operating characteristics (ROC) analysis was used to determine cut-off values that best discriminate two groups. The distribution of variables was assessed with Kolmogorov-Smirnow and Shapiro-Wilk normality tests. Nonparametric variables were log-transformed before their inclusion in regression models. Multivariate forward linear regression was performed to identify relationships between anti-CarP Ab and clinical parameters related to skin involvement in SSc. Most statistical tests were performed using SPSS v.21 for Windows. The visual binning feature in SPSS was used to divide SSc patients by age at observation (equal percentile binning; 4 cut-points, 20% width). ROC analysis was performed using MedCalc software (v. 7.6.0.0), and Youden’s index was used to obtain the optimal cut-off for anti-CarP Ab. For all tests, a p value <0.05 was considered significant.

## Results

### Antigen specificity

**Fig 1:**
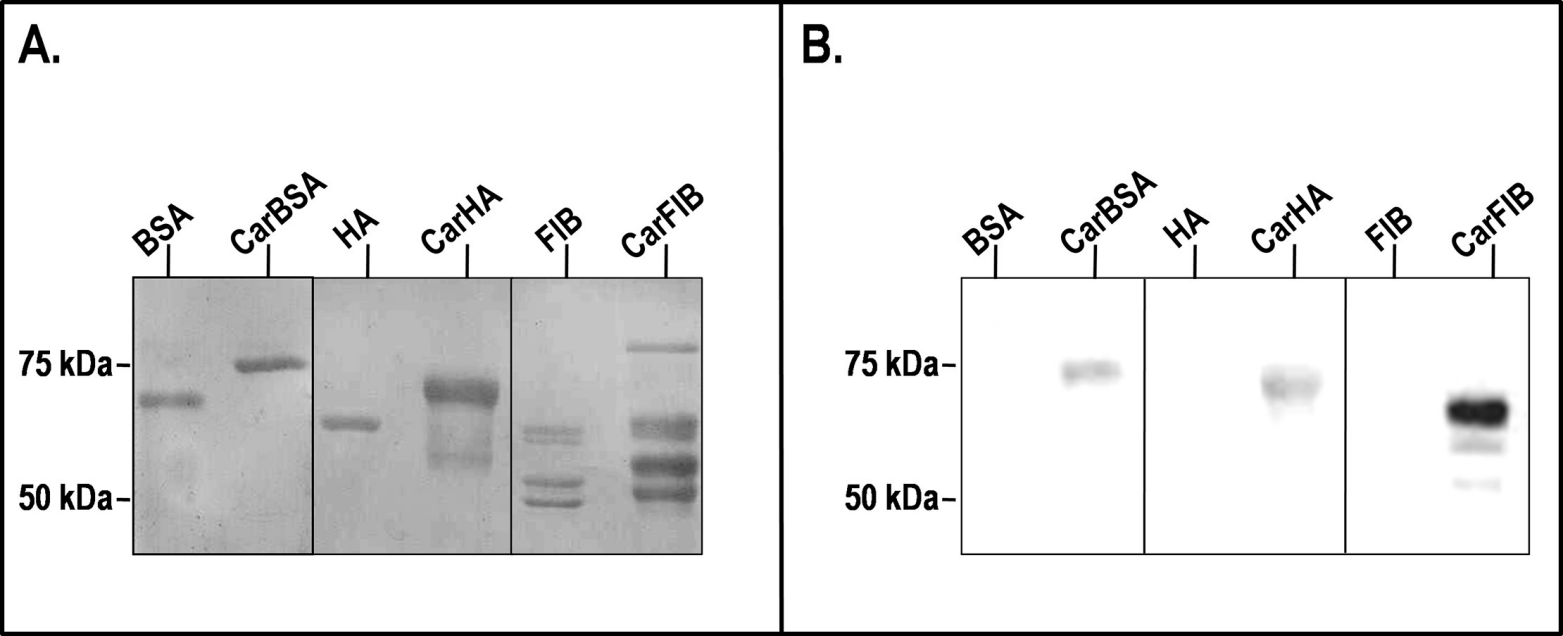
Electrophoretic mobility of carbamylated serum proteins and their immunoreactivity with anti-CarP Ab. (A) Native and carbamylated proteins (2 μg per lane) were analyzed by 10% SDS-PAGE under reducing conditions and stained with Coomassie brilliant blue. Molecular weight markers were run on a parallel track. (B) Native and carbamylated proteins (500 ng/lane) were separated by 10% SDS-PAGE under reducing conditions, transferred to a PVDF filter, and incubated overnight with affinity-purified anti-CarP Ab. Bound Ab were detected using HRP-conjugated goat anti-human IgG and ECL substrate. *BSA*, bovine serum albumin; *HA*, human albumin; *FIB*, fibrinogen; *Car*, carbamylated.

To characterize the specificity of anti-CarP Ab in SSc, we first evaluated whether the recognition of carbamylated epitopes depends on the species of origin or the type of target protein. Three serum proteins, namely bovine and human albumin (BSA and HA, respectively) and the structurally unrelated human fibrinogen (FIB), were studied. SDS-PAGE of the proteins, untreated and after the carbamylation procedure, revealed that the carbamylated forms exhibited slower electrophoretic mobility than their native counterparts, indicating successful carbamylation (**Fig 1A**). In the case of fibrinogen, carbamylation occurred on all three of its subunits, as indicated by changes in apparent molecular weight of α, β and γfrom ≈67, 54, and 47 kDa to ≈70, 59, and 51 kDa, respectively. To test the immunoreactivity of these proteins, we isolated anti-CarP Ab from a pool of SSc sera by affinity chromatography on a CarBSA-conjugated column, and used these Ab in western blotting. Anti-CarP Ab reacted with all the three carbamylated proteins tested (**Fig 1B**). For fibrinogen, the anti-CarP Ab mostly reacted with the α chain. In all cases, the reactivity was specific, since the anti-CarP Ab did not react with the corresponding native proteins used as negative controls.

**Fig 2:**
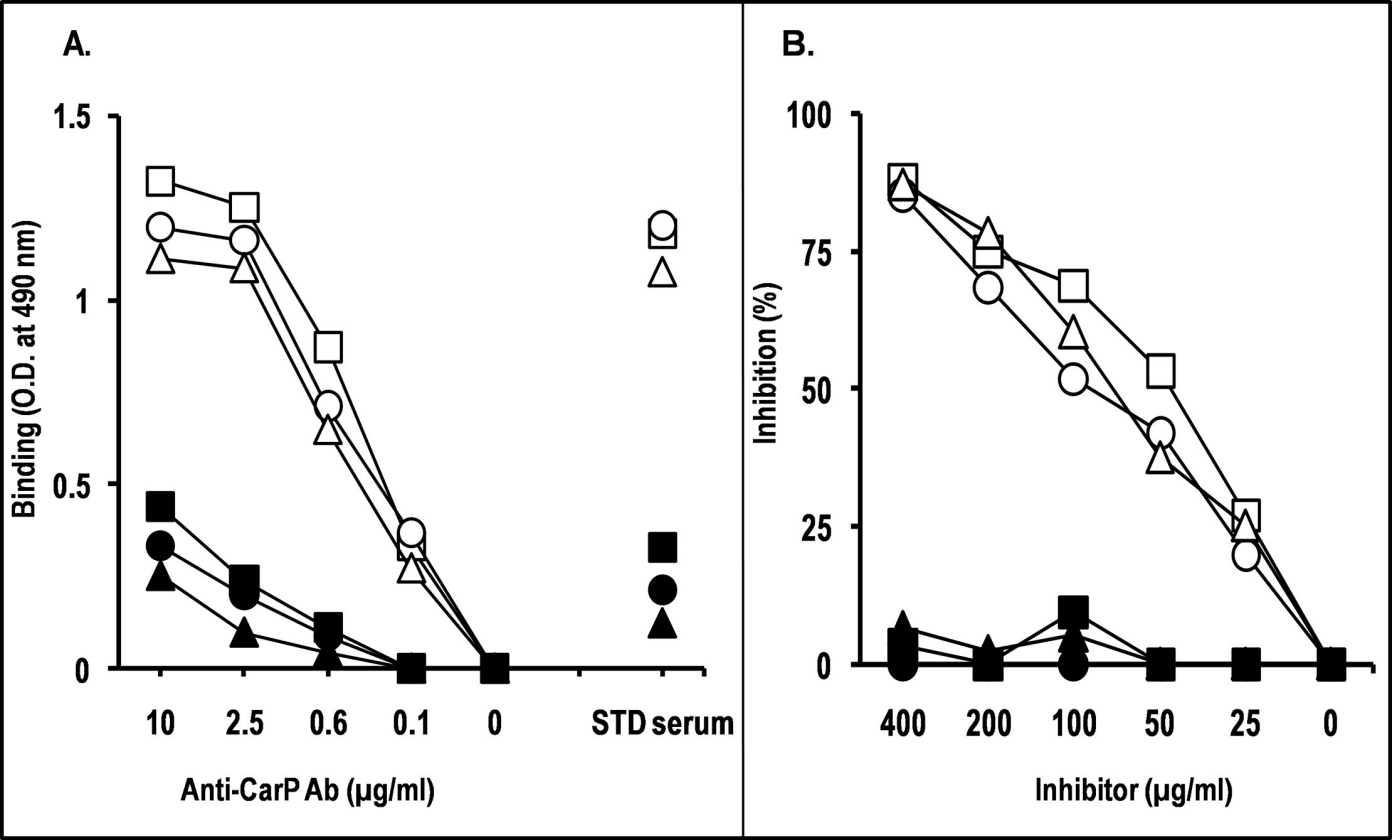
Specificity of anti-CarP Ab assessed by binding and inhibition ELISAs. (A) In the binding ELISA, microtiter plates were coated with BSA (square), HA (circle), or FIB (triangle), in carbamylated form (empty symbols) or native form (filled symbols). Wells were incubated PBS containing serial dilutions of anti-CarP Ab. Standard (STD) serum was used as specificity control. Bound IgG was revealed with HRP-conjugated anti-human IgG and *o*-phenylenediamine. The data are representative of at least two independent experiments. (B) In the inhibition assay, anti-CarP Ab were diluted in PBS-BSA to the lowest concentration giving 80%–100% of maximal A_490_ in the binding assay (2.5 μg/ml), and pre-incubated with an equal volume of PBS containing 2-fold serial dilutions of BSA (square), HA (circle), or FIB (triangle), in carbamylated form (empty symbols) or native form (filled symbols). Following a 2-h incubation at room temperature, the mixture was added to microtiter plate wells coated with CarBSA. After a 4-h incubation at room temperature and three washes, bound IgG was detected with HRP-conjugated anti-human IgG (Fc portion) and *o*-phenylenediamine. Results are expressed as percentage of binding inhibition compared with binding in the absence of inhibitor. The data are representative of at least two independent experiments.

To further investigate the specificity of the affinity-purified anti-CarP Ab, their ability to cross-react with different carbamylated proteins was analyzed by ELISA. Anti-CarP Ab reacted with CarBSA, CarHA and CarFIB to similar extents and in a dose-dependent manner (**Fig 2A**). The binding was specific since they did not react with the native proteins, with only some weak non-specific background binding being observed. When the ability of carbamylated proteins to inhibit anti-CarP Ab binding to CarBSA was evaluated, we observed that CarBSA, CarHA, and CarFIB inhibited anti-CarP Ab binding to similar extents (**Fig 2B**). The inhibition was dose-dependent and specific, since unmodified proteins did not affect the binding. These results suggest that the binding between anti-CarP Ab and the epitope occurs independently of protein type or species origin.

### Clinical associations of Anti-CarP Ab in SSc

To evaluate whether anti-CarP Ab are associated with skin involvement in SSc, we investigated 124 SSc patients (**Table 1**). For all the analyzed parameters, the percentage of missing values was below 4%. The SSc patients were predominantly female (95.2%), their mean age was 53.8 years, and mean disease duration was over 14 years. As evidenced by the disease severity scale, the most affected organ systems were the peripheral vascular system (mean score, 1.56) and the lungs (mean score, 1.51). The mean mRss was 8.69, while 50 cases (40.2%) had scleredema (score >0). The distribution of scores by organ system is shown in **Table 2.**

**Table 1:** Characteristics of the 124 patients with systemic sclerosis included in the study.

Variable	Value
Female, n (%)	118 (95.20)
Age at diagnosis^a^, mean (SD), years	39.71 (15.56)
Age at observation, years	53.80 (12.42)
Disease duration (time since RP, years)	14.09 (10.21)
Disease severity scale items, mean (SD)	
General	0.51 (0.79)
Peripheral vascular	1.56 (0.80)
Skin	1.11 (0.70)
Joint/tendon	0.47 (1.06)
Muscle	0.40 (0.68)
Gastro-intestinal tract	0.89 (0.49)
Lung	1.51 (1.11)
Heart	0.25 (0.66)
Kidney	0.10 (0.59)
Total score	6.77 (3.63)
mRss, mean (SD)	8.69 (8.35)
Scleredema>0, n (%)	50 (40.20)
Smoker, n (%)	6 (4.83)

mRss, modified Rodnan skin score; RP, Raynaud's phenomenon; SD, standard deviation.

^a^Onset of the first Raynaud’s phenomenon

**Table 2:** Disease severity scores in patients with systemic sclerosis.

Organ system	0 (Normal)	1 (Mild)	2 (Moderate)	3 (Severe)	4 (End stage)	Total
General	75	39	5	2	2	123
Peripheral vascular	2	70	31	19	1	123
Skin	20	76	23	5	0	124
Joint/tendon	97	8	69	3	6	123
Muscle	85	27	8	2	0	122
Gastro-intestinal tract	20	95	3	2	0	120
Lung	29	28	41	19	4	121
Heart	102	9	6	3	0	120
Kidney	118	1	1	1	2	123

Values are the number of patients with systemic sclerosis.

**Fig 3:**
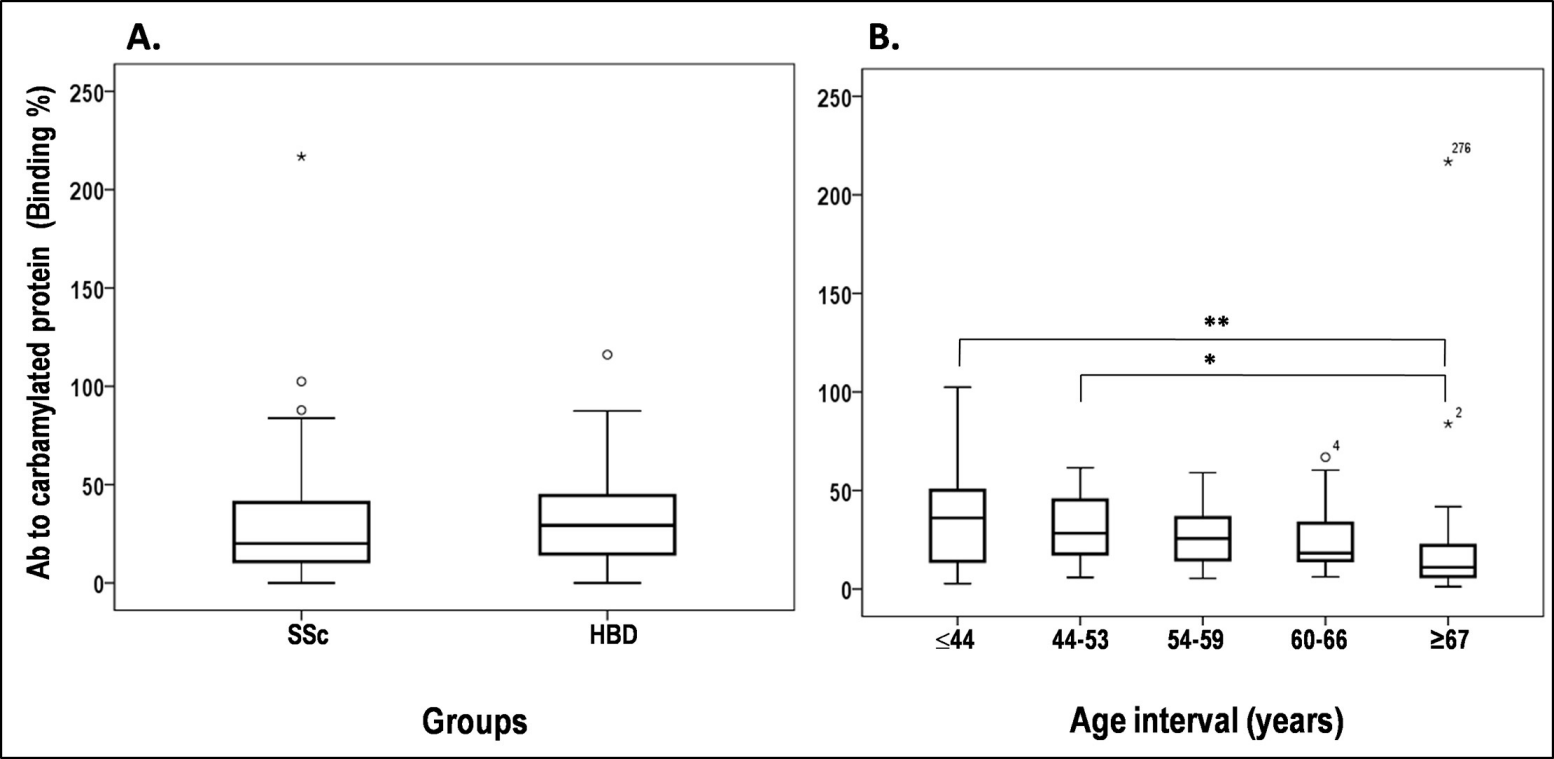
Levels of anti-CarP Ab in SSc patients and healthy blood donors. (A) Sera from 124 SSc patients and 41 healthy blood donors were screened for specificity to CarBSA by indirect ELISA. Binding of anti-CarP Ab is expressed as a percentage of the binding obtained with positive control serum from an RA patient. (B) Anti-CarP Ab levels in SSc patients divided by age at observation into five groups: I, ≤44 years, 32 cases; II, 44–53 years, 28 cases; III, 54–59 years, 24 cases; IV, 60–66 years, 17 cases; and V, ≥67 years, 23 cases. Horizontal bars mark the medians and boxes indicate interquartile ranges; outliers (more than 1.5 times the interquartile range) are marked with circles, while extreme outliers (more than 3 times the interquartile range) are marked with asterisks. Kruskal-Wallis Htest, *p = 0.037, **p = 0.014.

The levels of anti-CarP Ab in SSc patients were determined by indirect ELISA and compared with that of 41 age-matched HBD. This analysis showed no significant difference in the mean levels [± standard deviation] of anti-CarP Ab between SSc (28.17±25.43) and HBD (34.24±24.47) groups (**Fig 3A**). To evaluate the impact of age on anti-CarP Ab serum levels, SSc patients were divided into five groups on the basis of age (**Fig 3B**). Only group V had median anti-CarP Ab levels (11.02±45.54) significantly lower than those of group I (36.05±24.26; Kruskal-Wallis p<0.001) and group II (28.30±16.80; p = 0.037), suggesting a trend of age-dependent influence on anti-CarP Ab levels. No differences in median levels were observed among the other groups.

Spearman's correlation analysis was performed to define the relationship between serum anti-CarP Ab levels and age or mRss in SSc patients. As reported in **Table 3**, anti-CarP Ab levels were inversely associated with the age at observation (R = -0.27, p = 0.002) and with mRss (R = -0.32, p<0.001). Because lower anti-CarP Ab were observed in the oldest age group (≥67 years), we repeated Spearman's analysis after group V exclusion. Even with this exclusion, the association between anti-CarP Ab and mRss remained statistically significant (p = 0.008), suggesting that the inverse association between anti-CarP Ab and mRss is not influenced by age. For this reason, we decided to use the whole population for further investigations.

**Table 3:** Antibodies (Ab) to carbamylated BSA (CarBSA) inversely correlate with age and modified Rodnan skin score in patients with systemic sclerosis.

Variable	Age group	Patients, n	Spearman’s R	p
Age at observation	Any	124	-0.27	0.002
	<67	101	-0.15	0.110
mRss	Any	120	-0.32	<0.001
	<67	99	-0.27	0.008

mRss, modified Rodnan skin score. A p<0.05 was considered statistically significant.

We also assessed a possible relationship between anti-CarP Ab and SSc-specific auto-Ab, namely ACA and anti-Scl70 Ab. This analysis revealed no significant differences in the mean levels [± standard deviation] of anti-CarP Ab between patients who were ACA^+^ (25.72±18.93)and patients with anti-Scl70 Ab (28.37±19.51; Mann-Whitney p = 0.539).

**Fig 4:**
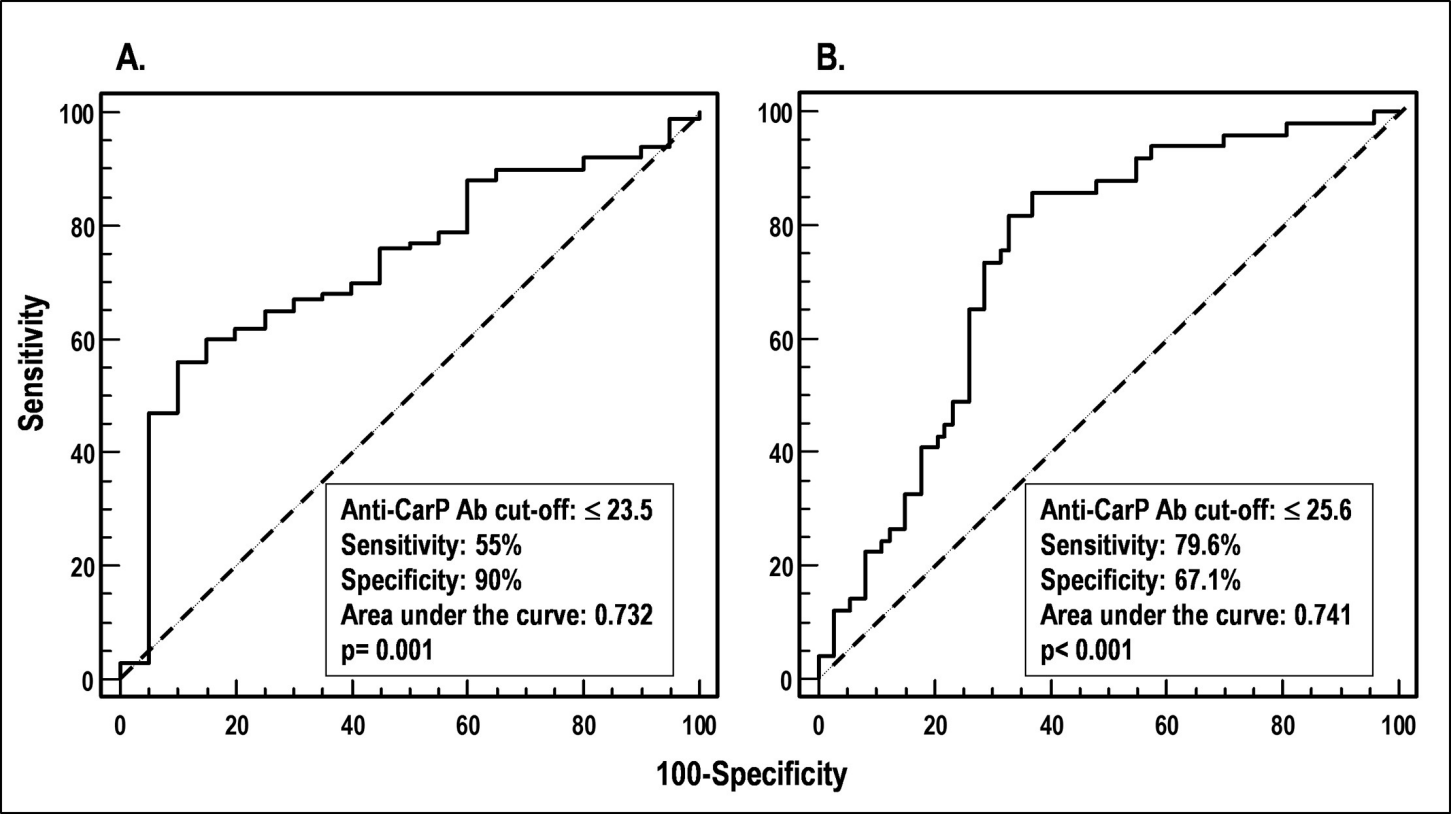
Receiver operating characteristic (ROC) analysis to define levels of anti-CarP Ab (cut-offs) that distinguish patients according to clinical variables. (A) SSc patients with mRss>0 *vs* those with mRss = 0. (B) SSc patients with scleredema *vs* those without it.

ROC curves were generated for anti-CarP Ab to evaluate their ability to discriminate dichotomized clinical variables describing skin involvement in SSc. Acceptable ROC curves (p<0.05) were obtained only with the dichotomization of SSc patients according to mRss (**Fig 4A**) and to the presence or absence of scleredema (**Fig 4B**). The best anti-CarP Ab cut-off discriminating patients with mRss>0 (versus those with mRss = 0) was ≤23.5% of the binding (AUC = 0.732, p = 0.001, 55% sensitivity, 90% specificity), while the best cut-off discriminating patients with scleredema from those who did not have it was ≤25.6% (AUC = 0.741, p<0.001, 79.6% sensitivity, 67.1% specificity). These results indicate that anti-CarP Ab are inversely associated with worse skin involvement in SSc patients.

Finally, to further analyze the relationships between anti-CarP Ab and skin involvement and its interdependency with age, multivariate forward linear regression was performed by including mRss (score 0 *vs*>0) and scleredema (absence *vs* presence) as outcome variables, and anti-CarP Ab levels (log-transformed) along with sex, age at diagnosis, age at observation, disease duration (log-transformed) and clinical type (limited or diffuse) as predictor variables. As shown in **Table 4**, low levels of anti-CarP Ab were associated with mRss>0 and the presence of scleredema, indicating that these Ab inversely associated to the severity of skin involvement, independently of disease duration, both in the whole cohort and in the age group <67. Disease type (limited *vs* diffuse), sex, age at diagnosis and age at observation remained excluded from the model. When “age at observation” was used as the outcome variable, anti-CarP Ab levels were not retained in the model, supporting the lack of association between these two variables.

**Table 4:** Antibodies (Ab) to carbamylated BSA (CarBSA) are associated to a worse skin involvement.

Outcome variable	Age group	Predictor retained in the model	B	SE	p	OR	95% CI for OR
mRss	Any	Ab to CarBSA	-2.91	1.06	0.006	0.05	0.070–0.430
	<67	Ab to CarBSA	-2.70	1.14	0.018	0.07	0.007–0.630
Scleredema	Any	Ab to CarBSA	-3.20	0.76	<0.001	0.04	0.010–0.220
		Disease duration	-2.40	0.80	0.002	0.09	0.020–0.420
	<67	Ab to CarBSA	-3.00	0.88	0.001	0.05	0.010–0.281
		Disease duration	-2.24	0.84	0.008	0.10	0.020–0.554

CI, confidence interval; mRss, modified Rodnan skin score; OR, odds ratio; SE, standard error. A p<0.05 was considered statistically significant.

## Discussion

In the present study, we characterized the specificity of Ab directed against carbamylated proteins in SSc. We demonstrated that the recognition of carbamylated proteins by anti-CarP Ab occurs by means of the carbamylation-dependent epitope, irrespective of the species origin or type of protein.

The *in vitro* carbamylation of fibrinogen modified all its three distinct bands, but the anti-CarP Ab reacted strongly only with the α chain. Similar western blotting results have previously been reported by Shi et al., who used a different procedure to carbamylate fibrinogen and who tested anti-CarP Ab from RA sera [39]. The lack of immunoreactivity of the β and γ chains may be due to their intrinsic properties (i.e. fewer, less accessible lysine residues) or, alternatively, to the conformation of their carbamylated epitopes. If the latter possibility is the case, then both linear and conformational epitopes are generated by carbamylation.

In this study, we found no differences in serum levels of anti-CarP Ab between SSc patients and normal healthy subjects. This finding is not unique, since similar results were obtained by Pecani et al. [21], who measured anti-CarP Ab levels in different autoimmune diseases including SSc. This observation contrasts, however, with reports of high levels of anti-CarP Ab in rheumatic disorders, including RA [19,31,40,41], systemic lupus erythematosus (SLE) [42,43], and Sjögren’s syndrome [44]. In these studies, Ab levels associated with disease activity (in RA [19], SLE[42,43] and Sjögren's syndrome [44]) and with bone erosion (in RA [45–47]), and were predictive of RA onset in anti-CCP-positive patients with arthralgia without arthritis [48]. The postulated mechanism underlying these findings is that anti-CarP Ab levels increase concomitantly to the increase in carbamylated protein triggered by inflammation caused by the disease [19,44].

We found a progressive, age-dependent decrease in anti-CarP Ab serum levels in our patient cohort, in line with the reported age-dependent accumulation of carbamylated dermal collagen [49]. The decrease was modest, as only patients in the ≥67 years age group had median anti-CarP Ab levels significantly lower than those of patients in the lowest age groups (≤44 years and 44–53 years), irrespective of their clinical conditions. Even so, it is unlikely that age itself influenced these Ab levels, for the following reasons: 1) the correlation between anti-CarP levels and mRss remained statistically significant after the exclusion of patients aged ≥67 years; 2) the predictor variables "age at diagnosis" and "age at observation" were not retained in the model having mRss as an outcome variable; and 3) anti-CarP Ab levels were not retained in the model using "age at observation" as outcome variable.

The mechanisms underlying the associations between low anti-CarP Ab levels and the severity of skin involvement, reflected by mRss>0 and scleredema, can only be speculated at the present. One possibility is that high amounts of carbamylated proteins in skin, the target of carbamylation[49], may act like a sponge that soaks up circulating anti-CarP Ab, neutralizing them and significantly reducing their serum levels. If this is the case, then another mechanism of anti-CarP Ab level regulation should be considered besides that already hypothesized for RA, SLE and Sjögren’s syndrome, namely that Ab levels rise in response to disease activity-dependent neo-CarP epitope formation [19,44]. This issue may be resolved by analyzing the expression of carbamylated proteins in skin biopsies from SSc patients with different extents of dermis involvement in parallel with measuring anti-CarP Ab serum levels. The identification of biomarkers associated with skin involvement sets the ground for future research evaluating their potential usefulness in the prognosis and risk stratification of different cohorts of SSc patients.

Finally, all anti-CarP Ab ELISAs performed so far [21, 25], like our own, used polyclonal anti-CarP Ab that were prepared in-house and thus are not suitable for worldwide use in clinics and laboratories. There is, therefore, a great need for a human monoclonal Ab specific for carbamylated protein that would allow anti-CarP determinations to be done in a standardized manner. Experiments along this line are ongoing in our lab.
